# Degradation of Tocopherol Molecules and Its Impact on the Polymerization of Triacylglycerols during Heat Treatment of Oil

**DOI:** 10.3390/molecules24244555

**Published:** 2019-12-12

**Authors:** Dominik Kmiecik, Monika Fedko, Aleksander Siger, Bartosz Kulczyński

**Affiliations:** 1 Department of Gastronomy Science and Functional Food, Faculty of Food Science and Nutrition, Poznan University of Life Sciences, Wojska Polskiego 31, 60-634 Poznan, Poland; monika.fedko@up.poznan.pl (M.F.); bartosz.kulczynski@up.poznan.pl (B.K.); 2 Department of Biochemistry and Food Analysis, Faculty of Food Science and Nutrition, Poznan University of Life Sciences, Wojska Polskiego 31, 60-634 Poznan, Poland; aleksander.siger@up.poznan.pl

**Keywords:** tocopherols, triacylglycerol polymers, thermal decomposition of oil, heat treatment, pressed and refined oils, surface area to volume ratio, size-exclusion chromatography (HPSEC)

## Abstract

The aim of the study was to analyze the influence of the surface area to volume ratio of pressed and refined rapeseed oils on the changes in tocopherol content and polymerization of triacylglycerols during heating. In the study the pressed and refined rapeseed oil was heated at 170 °C, during 6, 12, and 18 h with three different surface area to volume (s/v) ratios (0.378, 0.189, and 0.126 cm^−1^). During heating, a decrease in tocopherols and increases in dimers, trimers, and oligomers of triacylglycerols were observed. However, the changes were dependent on the surface area to volume ratio used, type of oil and time of heating. The biggest changes were observed in oil with the biggest s/v ratio (0.378 cm^−1^), and the lowest when the s/v ratio was 0.126 cm^−1^. The pressed oil was characterized by faster degradation of tocopherols and slower increase of triacylglycerol polymer levels compared to refined oil.

## 1. Introduction

Fried food is one of the most popular types of food consumed worldwide. By the frying process, we can obtain the elements of the main dishes and different types of side dishes such as French fries or other fried potato products. Additionally, on the market, we can find a different type of pastries or salty snacks, wherein the preparation step—the frying process—played a major role. The frying process is present in restaurants and household kitchens, catering systems, industry (production of frozen fried potato products, extruded noodles), and street food points of sale. The popularity and magnitude of fried food consumption have two reasons: (I) sensory (texture, color, and flavor) and (II) economic (the frying process is easy to use, fast, and relatively cheap). During frying, the food is immersed and cooked in hot oil, and the high temperature of the process (150–200 °C) leads to irreversible changes in food. However, as a result of the processes which occur during frying (dehydration of food, Maillard reactions), fried food is characterized by unique sensory properties such as golden color, characteristic flavors and crispy texture [1,2]. The food is immersed in oil for a short time, while the oil used in frying is often exposed to high temperatures for a long time. During frying, there are three main factors influencing oil quality: oxygen from the air, moisture from foodstuff and the temperature of the process. Because of these factors, the oil undergoes undesirable transformations as a result of oxidation, hydrolysis and thermal reactions [3]. Oxidation is one of the first reactions that can be observed during oil heating. As a result of oxidation in the heated oil, both lower and higher molecular weight compounds compared to the parent triacylglycerols are formed. Oxidized fatty acids, aldehydes, ketones, hydrocarbons, alcohols, or epoxides are lower molecular weight compounds formed during the oxidation of triacylglycerols. The oxidation process, as a result of the free radical mechanism, also leads to the formation of dimers, trimers, and oligomers of triacylglycerol (TAG polymers) with higher molecular mass [4]. During the radical reaction, polar and non-polar dimers with C-O-C, C-O-O-C or C-C bridges in their structure are formed. TAG polymers can also be created during polymerization according to the non-radical acid-catalyzed mechanism which is caused by the high temperature of the frying process [2]. The radical and non-radical mechanisms are the main ways to create polymers in oil. However, during frying, when the steam layer from food limits the oxygen access, the non-radical mechanism can play the main role in the polymerization process [5]. The speed of the oxidation process depends on many factors that are related to the type of used oil, the type of frying product and the conditions of the frying process. Factors that accelerate the oxidation process are high levels of unsaturated fatty acid, high temperatures, long time of use of the oil, the presence of prooxidants in fried food, or an increased surface area to volume (s/v) ratio [6,7,8,9]. Antioxidants which naturally occur or which are added to oils are the main factor limiting the oxidation process. Tocopherols are the main group of antioxidants naturally occurring in oils. However, other substances with antioxidant properties such as phenolic compounds, phytosterols or squalene also occur in the oil. The added antioxidants are divided into two groups: synthetic (butylated hydroxytoluene-BHT, butylated hydroxyanisole-BHA, *tert*-butylhydroquinone-TBHQ, gallates) and natural (tocopherols, phenolic compounds obtained as an extract from different plants) [10]. The antioxidant activity of natural or synthetic antioxidants were tested in different models system in the last few decades and show varying effects depending on the model (emulsions, bulk oil, food), source of antioxidants (extracts of plants, tocopherols, synthetic) and model conditions (temperature, time) [11,12,13,14].

During frying, the oxygen concentration over the surface of the oil and the oxygen access (surface area) had a big impact on the oxidation process and thermal degradation of oil [6,15,16]. However, these studies are mostly related to the content of volatile compounds formed during the oxidation of oils heated at high temperature. There is a lack of information about the influence of surface area or surface area to volume ratio of oil on the content and composition of polymers created in oil at elevated temperature. The increase of polymers is significant both from the technological point of view and the nutritional value of fried fats and fried food. A high content of polymers reduces the heat transfer process and increases the viscosity of used oil [17,18]. Both phenomena lead to an increased fat content in the resulting fried products. The polymers with higher molecular weight might affect epithelial cells and microflora metabolites in the colon, lipid blood profile, renal functions or peroxidation of lipids in the human body. The polymers from frying oil can also be the factor increasing the intestine oxidative stress which is very often connected with several lifestyle diseases [19,20,21]. The aim of this study was to therefore to analyze the influence of surface area to volume ratio of pressed and refined rapeseed oils heated in elevated temperature on the changes of tocopherol levels and the thermal polymerization of triacylglycerols.

## 2. Results and Discussion

### 2.1. Characteristic of Pressed and Refined Rapeseed Oil

Both the pressed (PRO) and refined (RRO) rapeseed oil used in the study were characterized by a similar fatty acids composition, iodine value, and total polar compounds content. The fatty acid composition was typical for rapeseed oil. The main group of these components was monounsaturated fatty acids (MUFAs), which accounted for more than 64% of all fatty acids (Table 1). The polyunsaturated fatty acids (PUFAs) were 29.45% and 28.96% in pressed and refined oil, respectively. The saturated fatty acids (SFAs) were 6.48% in pressed and 6.90 in refined oil. A lower level of total polar compounds (TPCs) content was found in refined oil (0.9%). However, the results were very close to the TPC content in pressed oil (1.0%). Differences between used oils in tocopherols and plastochromanol-8 (PC-8) content were observed. The refined oil was characterized by a higher content of tocopherols and PC-8. The total content of tocopherols in this oil was 77.14 mg/100 g and for PC-8 it was 5.00 mg/100 g. In pressed rapeseed oil, the total content of tocopherols was 62.29 mg/100 g and that of PC-8 was 4.42 mg/100 g. The main tocopherols in both oils were α- and ƴ-tocopherol. However, in pressed oil, the α- and ƴ-tocopherol accounted for 44.64% and 54.05% of the total content of tocopherols, respectively. The refined oil was characterized by a lower share of α-tocopherol (40.35% of total tocopherols) and a higher share of ƴ-tocopherol (58.48%). δ-Tocopherol in both oils accounted for about 1% of total tocopherols.

### 2.2. Total Polar Compounds (TPC) Content

The heating process led to an increase in the content of TPC in the analyzed samples (Table 2).

In all samples, the increase of TPC was connected with extended heating time and the increase of the surface-volume (s/v) ratio of the oil. After 6 and 12 h of heating, the highest content of TPC was typical for samples of refined oil. There was only one exception. The pressed oil heated 12 h with a 0.378 cm^−1^ surface-volume ratio was characterized by a higher level of these components (26.4%) compared to refined oil (23.1%). The highest content of the TPC in samples after 18 h of heating and with the highest ratio (0.378 cm^−1^) were observed. However, in pressed oil, the TPC content was higher (40.3%) than in refined oil (37.2%). The highest content of TPC in pressed oil after 18 h of heating was also observed in oil with 0.189 cm^−1^ s/v ratio. The final level of polar material was 31.2% and 26.6% in pressed and refined oil, respectively. When oil was heated with 0.126 cm^−1^ s/v ratio the opposite situation was observed.

The highest TPC level was found in refined oil. In pressed oil, the prolongation of the heating time led to a steady increase in the TPC content in all samples. In refined oil, the highest increase of TPC content was observed during the first 6 h of heating. In samples with 0.378 and 0.189 cm^−1^ s/v ratio, the TPC content was 17.2, 16.2 and 12.4%, respectively. These values were 46%, 61% and 53% of the final content of the polar fraction, respectively. A high content of polar compounds during frying or heating of oil was also observed by other authors [7,22,23,24,25]. However, the results presented in many publications very varied. During 16 h frying of French fries in rapeseed oil, an increase in TPC above 45% was observed [22]. The authors observed lower levels of these compounds when rapeseed oil was used to prepare oil blends with more stable oils (corn, palm, or olive oil). The lowest level (25%) was found in the mixture of rapeseed oil and palm olein. A decrease in the content of polar compounds formed during heating was also observed when the layer thickness of the heated oil increased [7]. The time needed to reach the TPC limit level (25%) increased almost five times (from 71 to 315 min) with an increase in the thickness of the oil layer from 0.5 to 2.5 cm. 

The increase of the TPC content in heated oil is a common phenomenon and determines the total undesirable changes of oil during frying. The high temperature of the process, the stream from food, and the oxygen from air lead to the formation of many compounds, especially ones of a polar nature [21,26]. The time of use of the oil and the ratio between the surface and volume of oil also play a very important role in the final TPC content level. These two phenomena can be observed in Table 2. The TPC content increased with increasing heating time and decreased with decreasing surface area to volume ratio. The differences between oil can result from the initial content of antioxidants in unheated oil. The pressed oil was characterized by a lower content of tocopherols compared to refined oil (Table 1). However, the TPC content in this oil after 6 and 12 h was lower compared to refined oil. The pressed oils contain not only the tocopherols but also a higher content of phenolic compounds, which can be strong antioxidants in elevated temperature [2,12,27]. The use of phenolic compound-rich extracts from *Sorbus aucuparia* (L.) and *Malus baccata* (L.) berries during frying led to a significant reduction in the TPC content compared to the control sample (without addition) and to the oil test with the addition of the synthetic antioxidant BHT [28]. Similar or stronger properties were also observed when rapeseed oil was heating with the addition of extracts obtained from tea leaves [12].

The refining process decreases the content of tocopherols and phenolic compounds in the final products [29,30]. The highest content of TPC in pressed oil after 18 h of heating can result from degradation of these compounds during the first 12 h of heating and the lower initial content of the tocopherols. The highest content of polar material almost in all samples of refined oil may also result from a pro-oxidative role of triacylglycerol polymers present in the heated oil [31]. The authors heated the purified and unpurified oils in a Rancimat device, observing a reduction of the induction times of tested oils. The increase of the content of triacylglycerol polymers in samples of oil accelerated the oxidation process. The total content of triacylglycerol polymers in refined oil in this study was always higher compared to pressed oil (Table 3).

### 2.3. Tocopherols Content

**Figure 1:**
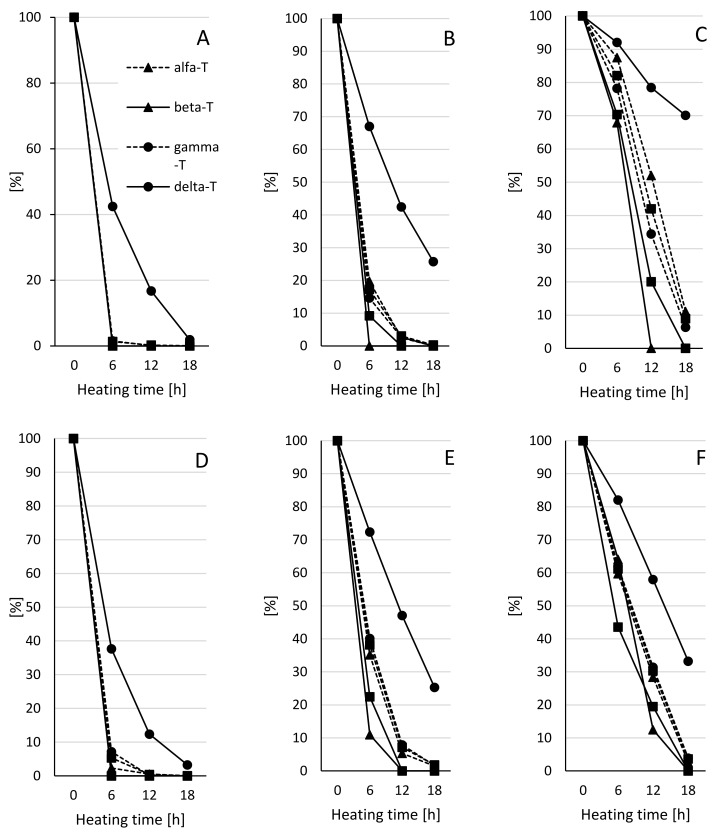
Changes in the content of tocopherols [%] during heating pressed and refined rapeseed oil heated at 170 °C with different surface area to volume (s/v) ratio. ABC-Pressed rapeseed oil, DEF-refined rapeseed oil. Ratio 0.378 cm−1 (**A**,**D**), 0.198 cm−1 (**B**,**E**), 0.126 cm−1 (**C**,**F**). T-tocopherol, PC-8-plastochromanol 8.

During heating, a decrease of tocopherol content was observed in all samples. However, the rate of degradation of these compounds depended on the surface area to volume ratio, time of heating, and type of oil (Figure 1). The ratio between surface area and volume of oil had the biggest impact of the degradation rate of the tocopherols. The fastest degradation of tocopherols was observed in oil heated with 0.378 cm^−1^ s/v ratio (Figure 1A,D).

In these samples, the total content of tocopherols during the first 6 h of heating was reduced by 98.67% and 94.68% in pressed and refined oil, respectively. In both oils, the fastest degradation was characteristic for α-, β-, γ-tocopherols, and PC-8. The slowest degradation rate was characteristic for δ-tocopherol. In both oils, δ-tocopherol was observed in all analyzed samples. However, the fastest degradation was characteristic for the first 6 h of heating, and it was 57.58% and 62.35% in pressed and refined oil, respectively. The final level of δ-tocopherol, after 18 h of the process, was 1.87% in pressed and 3.24% in refined oil of the content of this component in not heated oil. When the surface area to volume ratio was 0.189 cm^−1^, also the fast degradation of tocopherols and PC-8 was observed (Figure 1B,E). In pressed oil, after 6 h of heating, only β-tocopherol was degraded. Total degradation of α-, and γ-tocopherols, and PC-8 after 18 h of the process was observed. As before, the slower degradation of tocopherols and PC-8 was characteristic in samples of refined oil. When the refined oil samples were analyzed, only the total degradation of β-tocopherol and PC-8 was observed. These two compounds were degraded after 12 h of heating. In both oils, the fastest degradation of all compounds was observed during the first 6 h. After the first 6 h of the process, the total content of tocopherols was reduced by 82.72% and 61.79% in pressed and refined oil, respectively. The δ-tocopherol was the component that was degraded the slowest compared to others. After 18 h of heating, the content of δ-tocopherol was more than 25% of its content in unheated oil. The slowest degradation of tocopherols and PC-8 was observed when oils were heated with 0.126 cm^−1^ s/v ratio (Figure 1C,F). Additionally, in this case, the fastest degradation of tocopherols, comparing both oils, in refined oil was observed. In pressed oil, the lowest degradation rate of tocopherols was observed, during the first 6 h of the process. The total content of these compounds after the first step of heating was reduced by only 17.93%. At the same time, the reduction of the total content of tocopherols in refined oil was 38.85%. The final level of tocopherols in both oils was 90% less than the content in unheated oil. The total reduction of tocopherols during the heating process was 91.10% and 96.37% in pressed and refined oil, respectively. δ-Tocopherol was the most stable component. However, the biggest differences were observed comparing the results to two other ratios (0.378 cm^−1^ and 0.189 cm^−1^). Earlier, the degradation of δ-tocopherol was almost similar in pressed and refined oil. When the surface area to volume ratio was 0.126 cm^−1^, the degradation rate of this component was more than 2 times slower in pressed oil. After 18 h of heating, the total reduction of δ-tocopherol was 29.92% and 66.76% in pressed and refined oil, respectively.

The tocopherol degradation while using oils for frying is a common process that is most closely related to the temperature and time of process and type of used oil [32,33]. Of course, increasing the temperature and prolonged heating time leads to accelerated degradation of tocopherols in the oil. The effect of the chemical composition of the used oil is more complicated. The rate of tocopherol degradation depends not only on the degree of unsaturation of the oil. The main role is played by the tocopherol content in the oil and the proportions of individual tocopherols [34]. In rapeseed oil α-, β-, γ-, and δ-tocopherol were found. The highest biological activity is characteristic for α-tocopherol, but during frying, this compound was degraded the fastest [33,34]. Slower degradation rates for γ- and δ-tocopherol in elevated temperature were observed. Despite the significantly lower content of δ-tocopherol in the oil, it was more stable during heating [34,35]. The differences in the rate of degradation of individual tocopherols are related to their bond dissociation energy (BDE) of the phenolic hydrogen. The BDE order for tocopherols is: α < β ≈ γ < δ with ΔBDE values = −11.3, −9.4, −8.9 and −7.3 kcal/mol, respectively [36]. The α-tocopherol with lower BDE compared to other tocopherols is the predominant chain-breaking antioxidant. The δ-tocopherols with higher BDE also participate in breaking the oxidation reaction chain but not as intensely as α- or γ-tocopherol. The authors of [35] also found that better protective properties had oils containing a mixture of tocopherols (α, γ, δ) compared to oils with the addition of only one or two tocopherols. The combination of a high content of γ- and δ-tocopherol and low content of α-tocopherol also led to faster degradation of all added tocopherols. However, the chips were characterized by better oxidative stability compared to oil with a lower level of γ-, and δ-tocopherol, and high level of α-tocopherol. The degree of unsaturation of oil also play an important role. The highest degradation of these compounds is typical for oils with a high content of saturated fatty acids [32,37]. This phenomenon is explained by the presence of highly unsaturated oils—polyunsaturated fatty acids— which participate in the oxidation process, just as importantly as tocopherols. In this study, the oils used had a similar fatty acid composition and different content of tocopherols. The higher content of tocopherols in refined oil can explain the slower degradation of tocopherols in this oil during heating with 0.378 cm^−1^ and 0.189 cm^−1^ s/v ratio. However, not when the oil was heated with 0.126 cm^−1^ ratio. Probably, in two first cases, the main factor influencing the changes in oil was the oxygen access. The large surface area with small volume leads to rapid changes regardless of the content of antioxidants. When the ratio was 0.126 cm^−1^ the volume of oil was 3 and 1.5 times more compared to previous heating processes with the same oxygen access (the surface area was the same). Reducing the surface to volume ratio limits oxygen access to the entire mass of oil and increases the total tocopherol content in the total mass. This probably slows down the oxidation of the tocopherols, especially during the initial phase of the heating process. The slower degradation of the tocopherols in the pressed oil may be due to the presence of other compounds with antioxidant activity. Pressed rapeseed oil can be very good source of phenolic acids, especially sinapic acid, and their derivatives [27]. Canolol, a decarboxylation product of sinapic acid, and their dimers and trimers, showed a protective role against oxidation during frying [38].

### 2.4. Polymers of Triacylglycerols (TAG) Content

Heating of oils led to the formation of products with higher molecular weights than single triacylglycerols. The polymers were present in most of the analyzed samples. However, their content and composition depended on the type of heated oil and the conditions during heating (Table 3). In both oils, the main polymer fraction was TAG dimers. In a polar fraction, dimers ranged from 86.2 to 99.77% and from 63.6 to 86.4% in pressed and refined oils samples, respectively. In a non-polar fraction, these compounds accounted for 89.3 to 100% of all polymers. In a polar fraction, trimers and oligomers of triacylglycerols were also found, but TAG trimers were only characteristic of all the oil samples heated with 0.378 cm^−1^ s/v ratio and samples heated 18 h with 0.189 cm^−1^ ratio. Non-polar fractions also contained triacylglycerol trimers. However, they were more often observed in pressed oil than in refined oil. In pressed oil, trimers were found in all samples heated with 0.378 cm^−1^ ratio and after 18 h of heating with 0.189 cm^−1^ s/v ratio. In refined oil, triacylglycerol trimers were only found in samples heated 18 h with 0.387 cm^−1^ ratio. The increase of the content of polymers was connected with the increase in the surface area to volume ratio and the extension of the heating time.

The highest content of individual polymers, as well as their total content, was seen in samples heated for 18 h at the highest surface area to volume ratio (0.378 cm^−1^). Additionally, the content of polymers in refined oil samples was higher than in pressed oil. The total content of polymers in pressed oil heated 18 h ranged from 104.46 to 238.38 mg/g of oil and depends on the ratio between the surface area and volume of oil. The highest increase of total polymer content was characteristic for oil samples heated with 0.378 cm^−1^ s/v ratio. The lowest increase was found when the oil was heated with 0.126 cm^−1^ ratio. When the ratio was the smallest (0.126 cm^−1^), the final content of polymers was 43.82% and 56.84% of polymers in the samples heated with 0.189 and 0.378 cm^−1^ s/v ratio, respectively. When the ratio was 0.189 cm^−1^, the total polymers after 18 h of heating was 77.1% of the polymer content in oil heated with 0.378 cm^−1^ s/v ratio. During heating, the highest increase of polymers content in all samples was characteristic of the first step of the process (first 6 h). After 6 h of heating in samples with 0.378 cm^−1^ and 0.189 cm^−1^ ratio, the increase in polymer content was constant throughout the heating time. When the ratio was smaller (0.126 cm^−1^), the increase of polymers was higher between 6 and 12 h of the process and smaller during the last 6 h of heating. In refined oil, the total content of polymers was higher than in pressed oil and after 18 h of heating ranged from 180.39 to 308.25 mg/g of oil. The same as earlier, the smallest and the highest increase of those components in oil heated were found with the smallest (0.126 cm^−1^) and the highest (0.378 cm^−1^) s/v ratio. When the ratio was 0.126 cm^−1^, the final content of polymers was 58.52% and 83.05% of polymers in the samples heated with 0.189 and 0.378 cm^−1^ s/v ratio, respectively. When the ratio was 0.189 cm^−1^, the total polymers after 18 h of heating was 70.47% of polymer content in oil heated with 0.378 cm^−1^. The highest increase of polymers was observed in the first 6 h of heating. In the next steps of the process, the increase in these compounds was lower but constant. The increase of polymer content in heated oil is one of the main reactions observed when using the oils in elevated temperature. The highest content of these components in oil heated with high surface area to volume ratio results from the large contact area of oil with oxygen and a small volume of heated oil. Under these conditions, the oxidation process is very fast. The reduction of the surface to volume ratio (increase in the oil volume or decrease in the oil surface) had a protective effect on the quality of the heated oil [7]. The surface area to volume ratio plays an important role in the polymerization process because the oxidation process is one of the major pathways for the formation of triacylglycerol polymers [2]. The content of antioxidants and their composition are also very important factors influencing the content of polymers. The addition of γ-tocopherol showed stronger anti-polymerization properties than the addition of α-tocopherol during the heating of sunflower oil [34]. The anti-polymerization properties of γ-tocopherol also increased with the increase of the amount of additive [39]. A higher protective role at high temperature conditions, compared to α-tocopherol, was also found for δ-tocopherol [35,40]. In the present study, the highest increase of polymers in refined oil with a higher content of individual and total of tocopherols were found. Lampi and Kamal-Eldin [39] found the opposite effect, however, the authors tested the properties of tocopherols in purified triacylglycerols, eliminating the possible effect of other natural antioxidants or anti-polymerization agent on the content of triacylglycerols polymers. The pressed rapeseed oil contains also the high content of phytosterols and phenolic compounds, such as sinapic acid and their derivatives [41]. The phenolic extract obtained from rapeseed or other plants had a strong antioxidant and anti-polymerization activity in the high temperature of the process [12,28,42]. These properties can be connected with less volatility of phenolic compounds, better solubility in high temperature, and formation the secondary antioxidants with different or complementary antioxidant activity [28].

### 2.5. Principal Component Analysis (PCA)

**Figure 2:**
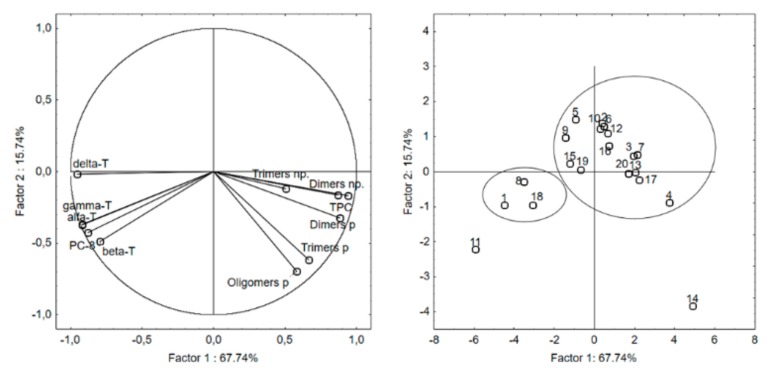
Principal component analysis (PCA) of the loadings plot and the score plot of data from α-, β-, γ-, and δ-tocopherols, plastochromanol-8 (PC-8), total polar compounds (TPC), and dimers, trimers and oligomers of triacylglycerol (TAG) in polar (p) and nonpolar (np) fraction of rapeseed oil heated at 170 °C.

Principal component analysis (PCA) was applied to observe possible clusters in the pressed and refined rapeseed oils heated at 170 °C with three different surface area to volume ratios. The first two principal factors accounted for 83.48% (PF1 = 67.74% and PF2 = 15.74%) of the total variation. The PCA results showed noticeable differences between the oils heated with the different surface area to volume ratio (Figure 2). Factor 1 was mainly correlated with the TPC content (r = 0.939), TAG dimers in the polar fraction (r = 0.883) and TAG dimers in the non-polar fraction (r = 0.871). It was also negatively correlated with the content of δ-tocopherol (r = −0.954), α-tocopherol (r = −0.919) and γ-tocopherol (r = −0.916). Factor 2 was mainly negatively correlated with the content of TAG oligomers (r = −0.696) and trimers (r = −0.615) in the polar fraction. The data showed in the score plot divided samples into two main groups. The small group, with samples 1, 8, and 18, located on the left side of the score plot, below the *x*-axis, contains samples with a low polymer content and high content of tocopherols and PC-8. These are samples of non-heated pressed oil and oils heated for 8 h at the lowest surface to volume ratio (0.126 cm^−1^). The second group contains the other samples of heated oils. However, samples located on the left side of the score plot (5, 9, 15, 19) were characterized by a low content of TAG dimers and trimers, the absence of TAG oligomers and mean content of tocopherols. On the right side of the score plot were located samples with a high and very high content of polymers and very low content of tocopherols. In the PCA score plot, there are two samples with a long distance from other groups. Sample 11 (non-heated refined rapeseed oil) were characterized by the lowest polymer content (0.08 mg/g of oil) and the highest content of tocopherols (77.14 mg/100 g) and PC-8. The second sample with a long distance from others was sample 14, refined rapeseed oil heated 18 h with 0.378 cm^−1^ surface to volume ratio. This sample was characterized by the highest content of polymers, more than 300 mg/g of oil.

## 3. Materials and Methods

### 3.1. Materials

Pressed (PRO) and refined (RRO) rapeseed oils were used in the study. The oils were purchased on the local market as commercial products at the same time. The main difference between the oils used during the study was the degree of processing. Pressed oil is obtained as a result of only mechanical treatment–pressing of seeds. Refined oil is an oil that undergoes other treatments in the production process after extraction or pressing of seeds (degumming, deacidification, bleaching, and deodorizing). These treatments lead to the removal of both desirable and undesirable substances found in oils. On the one hand, refined oils have a neutral taste and aroma, and have an extended shelf life. On the other hand, the refining process reduces the level of such compounds as tocopherols, sterols and phenolic compounds.

### 3.2. Heating Process

The oils were heated in glass beaker covered aluminum foil at 170 °C ± 5 °C in two parallel replications. The heating time was 6, 12 and 18 h and the volume of heated oil was 150 mL, 300 mL, and 450 mL. The ratio between the surface area and volume (s/v ratio) of oil was 0.378 cm^−1^, 0.189 cm^−1^ and 0.126 cm^−1^, respectively. The glass beaker with oil was heated using the magnetic stirrers (IKA RET basic, MS-H-Pro, IKA Works, Inc. Wilmington, NC, USA). The temperature was controlled throughout the heating using an electronic thermometer. At the end of the process, the oil samples were sealed under nitrogen and kept frozen at −24 °C until analysis.

### 3.3. Total Polar Compounds (TPC) Analysis

Total polar compounds content of oil was analyzed according to the American Oil Chemists’ Society-AOCS Official Method 982.27 [43]. Briefly, a sample of oil was dissolved in toluene and applied to a silica gel column (silica gel 60, 63–200 µm, Sigma-Aldrich, Poznan). A nonpolar fraction was eluted with a mixture of hexane and diisopropyl ether (82:18, *v:v*), and was collected. After evaporation of the solvent, the nonpolar fraction was weighted and from the weight difference of the sample and nonpolar fraction, the polar fraction was calculated. The results were expressed as % of the total content of the oil sample.

### 3.4. Iodine Value Calculation (CIV)

The iodine value of oil used for frying was calculated according to the AOCS Official Method Cd 1c-85 [44]. The method determines the iodine value directly from fatty acid compositions and based on the percentage of hexadecenoic acid, octadecenoic acid, octadecadienoic acid, octadecatrienoic acid, eicosanoid acid, and docosenoic acid.

### 3.5. Fatty Acid Composition Analysis

The fatty acid composition was determined according to AOCS Official Method Ce 1h-05 [45]. Briefly, oil was dissolved in hexane and transesterified with sodium methoxide. Fatty acid methyl esters (FAME) were analyzed using an Agilent 7820A GC system (Agilent Technologies, Santa Clara, CA, USA) equipped with a SLB-IL111 capillary column (100 m, 0.25 mm, 0.20 μm, Supelco, Bellefonte, PA, USA) and a flame ionization detector (FID). The FAME mixture was separated under the following conditions: the initial oven temperature was 150 °C, and it increased to 200 °C at 1.5 °C/min; the injector and detector temperature were 250 °C; split 1:10; the helium flow rate was 1 mL/min. The FAME were identified by comparison with commercially available standards–grain fatty acid methyl ester mix (Supelco). The results were expressed as the percentage of total fatty acids (see Supplementary Materials).

### 3.6. Tocopherol and Plastochromanol-8 Analysis

The tocopherols and plastochromanol-8 (PC-8) content were determined according to Siger et al. [46]. Briefly, oil was dissolved in *n*-hexane and transferred to vials for analyses. The tocopherol and plastochromanol-8 content were analysed using a Waters HPLC system (Waters, Milford, MA, USA) equipped with a LiChrosorb Si 60 column (250 × 4.6 mm, 5 µm, Merck, Darmstadt, Germany), a fluorimetric detector (Waters 474) and a photodiode array detector (Waters 2998 PDA). The mobile phase was a mixture of *n*-hexane with 1.4-dioxane (96:4, *v*:*v*). The flow rate was 1.0 mL/min (for tocopherols and PC-8). To detect the fluorescence of tocopherols and PC-8, the excitation wavelength was set at ʎ = 295 nm and the emission wavelength at ʎ = 330 nm. Standards of α-, β-, γ- and δ-tocopherols (>95% of purity) were purchased from Merck. The tocopherol standards were dissolved in n-hexane to obtain ten different concentration levels, in the range from 1 to 20 µg/mL, and injected to the HPLC system at 10 µL. The determination coefficient, which expresses the linearity of the standard curve, is equal to 0.99 and it is statistically significant (*p* < 0.0001) in case of all investigated standards. The limits of detection (LODs) for T were as follows: 0.86, 0.57, 0.64, 0.82, 0.89, 0.93, 0.75, 1.41, and 0.75 µg/mL for α-T, β-T, γ-T and δ-T respectively.

### 3.7. The Fractionation Into Polar and Nonpolar Fractions

An oil sample was fractionated into the polar and non-polar fractions using silica gel, according to Kmiecik et al. [47]. Briefly, the oil was dissolved in toluene and applied to a silica gel column. The non-polar fraction was eluted with a mixture of hexane and diisopropyl ether (82:18, *v*:*v*). The polar fraction was eluted with pure diisopropyl ether. The purity of both fractions and separation accuracy was verified with thin layer chromatography (TLC). A silica gel TLC plate was developed with hexane–diisopropyl ether (82:18, *v*:*v*), sprayed with a copper sulphate-phosphoric acid-methanol solution and heated at 120 °C.

### 3.8. Polymer Composition Analysis

The polymer composition was determined according to AOCS Official Method 993.25 [48] in the polar and non-polar fraction of oil. Briefly, 1 mL of the obtained oil fraction was transferred to a vial and then analysed by HPLC. The polymer composition was analyzed with an Infinity 1290 HPLC system (Agilent Technologies) equipped with an Evaporative Light Scattering Detector (ELSD) and two interconnected Phenogel columns (100Å and 500Å, 300 × 7.8 mm, 5 μm, Phenomenex, Torrance, CA, USA). The polymers were separated under the following conditions: column temperature 30 °C; detector temperature 30 °C; detector pressure 2.5 bars; injection sample volume 1 µL. The liquid phase was dichloromethane (DCM) and the flow rate was 1 mL/min.

### 3.9. Statistical Analysis

All assays were performed in two replications. Mean values and standard deviations were calculated with Microsoft Office Excel 2013 (Microsoft Corporation, Redmond, WA, USA). STATISTICA PL 13.0 (StatSoft, Inc., Cracow, Poland) was the software used for principal component analysis (PCA) and to calculate significant differences between the means (*p* < 0.05, analysis of variance ANOVA, Tukey’s multiple range test). The significance of differences between fresh oil samples was determined using the parametric student *t*-test (*p* < 0.05).

## 4. Conclusions

The study showed that the surface area to volume ratio of the heated oil had the biggest impact on the thermal changes of the oils and its components during frying. Reducing the surface to volume ratio leads to slower degradations of tocopherols and heated oils in a similar way. However, in pressed oil, the increase of polymers content was lower compared to refined oil. Lowering the surface to volume ratio three times led to a decrease in the content of polymers by 56% and 41% in the pressed and refined oil, respectively. The content of polymers of triacylglycerols in oil used for the frying process, and their reduction should be investigated. Our research shows how important the surface area to oil volume ratio is in the frying process. The selection of appropriate frying conditions can contribute to better tocopherol preservation and reduction of the content of undesirable triacylglycerol polymers.

## Figures and Tables

**Table 1:** Characteristics of fresh pressed (PRO) and refined (RRO) rapeseed oil.

	PRO	RRO
Fatty acid composition [%]		
16:0	4.32 ± 0.00a	4.76 ± 0.05a
18:0	1.54 ± 0.01a	1.56 ± 0.02a
18:1	63.57 ± 0.02a	63.83 ± 0.01a
18:2	18.74 ± 0.03a	19.07 ± 0.05a
18:3	10.70 ± 0.00a	9.89 ± 0.12a
20:0	0.54 ± 0.01a	0.47 ± 0.20a
20:1	0.32 ± 0.05a	0.06 ± 0.02b
22:1	0.18 ± 0.06a	0.26 ± 0.03b
24:0	0.08 ± 0.04a	0.11 ± 0.02a
Total SFA	6.48 ± 0.07a	6.90 ± 0.07a
Total MUFA	64.08 ± 0.01a	64.14 ± 0.19a
Total PUFA	29.45 ± 0.03a	28.96 ± 0.12b
Total Polar Compounds [%]	1.0 ± 0.1a	0.9 ± 0.0a
Iodine value	115.52 ± 0.06a	114.03 ± 0.12a
Tocopherols [mg/100 g]		
α	25.14 ± 0.26a	34.44 ± 0.26b
β	0.07 ± 0.03a	0.16 ± 0.01b
ƴ	36.43 ± 0.25a	41.70 ± 0.26b
δ	0.66 ± 0.06a	0.85 ± 0.03b
Total tocopherols	62.29 ± 0.59a	77.14 ± 0.04b
Plastochromanol-8	4.42 ± 0.04a	5.00 ± 0.05a

Values are means of two determinations ± SD. Means in the same row, followed by different small letters indicate significant differences (*p* < 0.05) between the pressed and refined oil.

**Table 2:** Changes of total polar compounds (TPC) during heating pressed (PRO) and refined (RRO) rapeseed oil heated with a different surface to volume of oil ratio [%].

Surface to Volume Ratio [cm^−1^]	Heating Time [h]			
	Not Heated	6	12	18
PRO				
0.378	1.0 ± 0.0aA	15.4 ± 0.5bA	26.4 ± 0.1cA	40.3 ± 1.1dA
0.189	1.0 ± 0.0aA	6.9 ± 0.1bB	17.9 ± 0.5cB	31.2 ± 0.4dB
0.126	1.0 ± 0.0aA	4.8 ± 0.9bC	12.3 ± 0.4cC	20.1 ± 0.2dC
RRO				
0.378	0.9 ± 0.0aA	17.2 ± 0.1bA	23.1 ± 1.8cD	37.2 ± 0.6dD
0.189	0.9 ± 0.0aA	16.2 ± 0.5bA	20.8 ± 0.1cE	26.6 ± 0.5dE
0.126	0.9 ± 0.0aA	12.4 ± 1.5bD	16.3 ± 0.1cB	23.5 ± 1.5dF

Values are means of four determinations ± SD. Means in the same row, followed by different small letters indicate significant differences (*p* < 0.05) between the same samples with different heating time. Means in the same column, followed by different capital letters, indicate significant differences (*p* < 0.05) between samples in the same heating time.

**Table 3:** The composition of a polar and nonpolar fraction of pressed (PRO) and refined (RRO) rapeseed oil heated at 170 °C with different surface area to volume (s/v) ratio [mg/g of oil].

**Time of Heating and s/v Ratio**	**Polar Fraction**				**Nonpolar Fraction**			**Total Polymers**
	**Monomers**	**Dimers**	**Trimers**	**Oligomers**	**Monomers**	**Dimers**	**Trimers**	
PRO								
Not heated	8.99 ± 0.06a	0.19 ± 0.04a	nd *	nd	962.21 ± 3.86m	nd	nd	0.19 ± 0.04a
6 h 0.378	109.94 ± 4.42de	18.65 ± 2.81bcd	1.91 ± 0.18ab	0.82 ± 0.14b	763.73 ± 8.43ij	28.03 ± 1.18b	3.37 ± 0.68b	52.78 ± 3.90d
12 h 0.378	150.26 ± 2.34g	51.36 ± 2.91fg	6.46 ± 0.90c	1.79 ± 0.20c	621.66 ± 3.05f	52.28 ± 3.14c	5.44 ± 0.78c	117.34 ± 1.87gh
18 h 0.378	215.99 ± 6.01i	122.49 ± 5.63k	14.09 ± 0.31e	3.54 ± 0.09d	455.59 ± 7.79b	92.87 ± 8.32f	5.39 ± 0.33c	238.38 ± 11.22k
6 h 0.189	47.39 ± 1.39b	10.06 ± 0.65b	0.03 ± 0.01a	nd	838.38 ± 8.87k	24.23 ± 2.09b	nd	34.32 ± 1.60c
12 h 0.189	110.58 ± 3.67de	47.29 ± 3.54ef	0.44 ± 0.08a	nd	691.05 ± 15.53gh	34.73 ± 2.03b	nd	82.46 ± 4.94e
18 h 0.189	177.49 ± 7.08h	101.32 ± 2.27j	5.10 ± 0.63c	0.09 ± 0.03ab	548.67 ± 7.67d	75.14 ± 2.93e	2.15 ± 0.26a	183.80 ± 4.31i
6 h 0.126	45.33 ± 3.51b	1.52 ± 0.20a	0.02 ± 0.01a	nd	911.51 ± 3.27l	4.02 ± 0.29a	nd	5.55 ± 0.19b
12 h 0.126	109.68 ± 2.76de	10.04 ± 1.37b	0.14 ± 0.03a	nd	817.98 ± 3.15k	21.74 ± 2.71b	nd	31.93 ± 4.02c
18 h 0.126	138.09 ± 5.36fg	38.55 ± 1.89e	0.46 ± 0.11a	nd	705.62 ± 6.52h	65.45 ± 4.95de	nd	104.46 ± 3.74fg
RRO								
Not heated	8.29 ± 0.00a	0.08 ± 0.00a	nd	nd	988.87 ± 0.49m	nd	nd	0.08 ± 0.00a
6 h 0.378	119.35 ± 5.26ef	27.73 ± 2.07d	4.24 ± 0.53bc	0.12 ± 0.03ab	671.55 ± 2.12g	94.01 ± 4.32f	nd	126.10 ± 4.63h
12 h 0.378	113.55 ± 8.46de	64.98 ± 4.59h	13.49 ± 1.29e	3.18 ± 0.47d	581.51 ± 16.64e	100.55 ± 8.99f	nd	184.89 ± 10.13i
18 h 0.378	155.07 ± 11.55g	126.37 ± 3.33k	44.20 ± 3.83h	14.71 ± 1.11e	425.41 ± 4.67a	120.79 ± 4.66g	2.18 ± 0.41a	308.25 ± 8.03l
6 h 0.189	94.97 ± 6.38cd	21.25 ± 1.12cd	5.96 ± 1.14c	nd	747.45 ± 7.26i	53.10 ± 6.70cd	nd	80.31 ± 7.59e
12 h 0.189	119.25 ± 2.30ef	51.34 ± 2.38fg	9.68 ± 0.29d	nd	643.04 ± 7.55f	67.33 ± 3.90e	nd	128.35 ± 5.98h
18 h 0.189	147.90 ± 4.62g	80.02 ± 5.41i	18.13 ± 0.75f	2.00 ± 0.42c	516.97 ± 13.68c	117.05 ± 7.87g	nd	217.21 ± 11.96j
6 h 0.126	78.11 ± 9.65c	11.28 ± 1.22bc	5.75 ± 1.36c	nd	786.35 ± 16.21j	27.09 ± 4.95b	nd	44.12 ± 6.52cd
12 h 0.126	95.50 ± 4.34cd	23.74 ± 2.03d	13.62 ± 0.75e	nd	696.04 ± 9.43gh	51.26 ± 2.65c	nd	88.62 ± 4.16ef
18 h 0.126	118.06 ± 10.11ef	59.96 ± 5.48gh	25.99 ± 0.56g	nd	579.38 ± 10.23e	94.44 ± 7.63f	nd	180.39 ± 3.50i

Values are means of four determinations ± SD. Means in the same column, followed by different small letters indicate significant differences (*p* < 0.05) between the samples. * nd–not detected.

## Supplementary Materials

The following are available online at https://www.mdpi.com/1420-3049/24/24/4555/s1.
