# Supplementary material for: Degradation of Tocopherol Molecules and Its Impact on the Polymerization of Triacylglycerols during Heat Treatment of Oil
Source: Molecules. 2019 Dec 12;24(24):4555. doi: 10.3390/molecules24244555 (PMC6943668; doi:10.3390/molecules24244555)
Supplement: Supplementary file 1 [file molecules-24-04555-s001.pdf]

molecules

MDPI

Supplementary Materials

Degradation of tocopherols molecules and its impact on the polymerization of triacylglycerols during heat treatment of oil

Dominik Kmiecik[1,*], Monika Fedko[2], Aleksander Siger[3], Bartosz Kulczyński[4]

[1] Department of Gastronomy Science and Functional Food, Faculty of Food Science and Nutrition, Poznan University of Life Sciences, Wojska Polskiego 31, 60-634 Poznan, Poland; dominik.kmiecik@up.poznan.pl
[2] Department of Gastronomy Science and Functional Food, Faculty of Food Science and Nutrition, Poznan University of Life Sciences, Wojska Polskiego 31, 60-634 Poznan, Poland; monika.fedko@up.poznan.pl
[3] Department of Food Biochemistry and Analysis, Faculty of Food Science and Nutrition, Poznan University of Life Sciences, Wojska Polskiego 31, 60-634 Poznan, Poland; aleksander.siger@up.poznan.pl
[4] Department of Gastronomy Science and Functional Food, Faculty of Food Science and Nutrition, Poznan University of Life Sciences, Wojska Polskiego 31, 60-634 Poznan, Poland; bartosz.kulczynski@up.poznan.pl

* Correspondence: dominik.kmiecik@up.poznan.pl

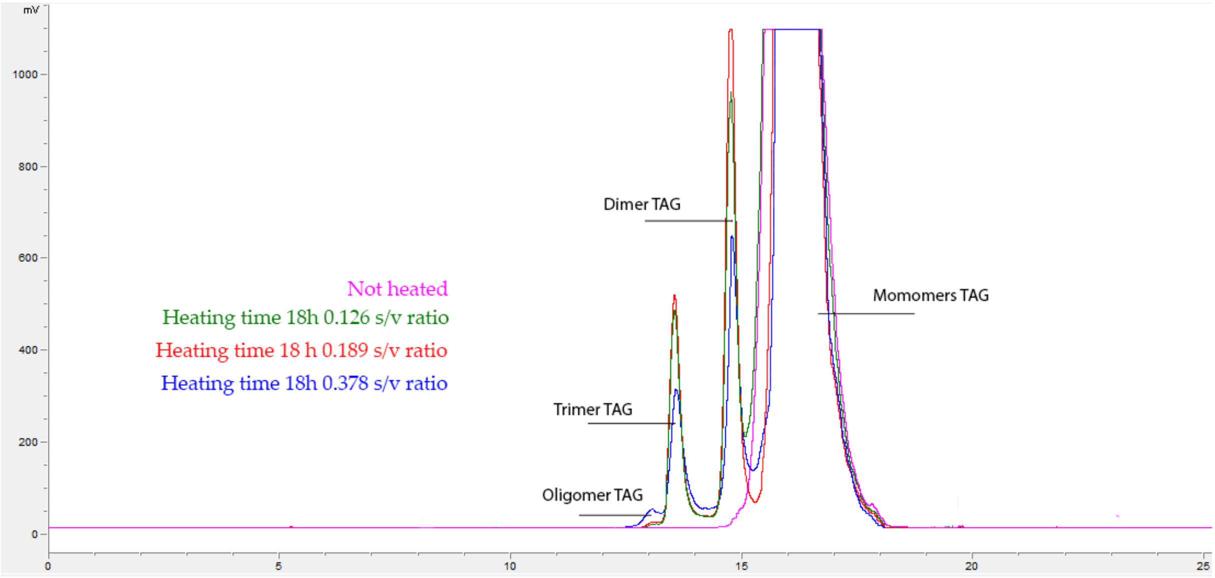

Figure S1. HPLC chromatogram of triacylglycerols polymers composition changes in rapeseed oil heated with a different surface area to volume ratio (0.378, 0.189, and 0.126 cm^{-1}).

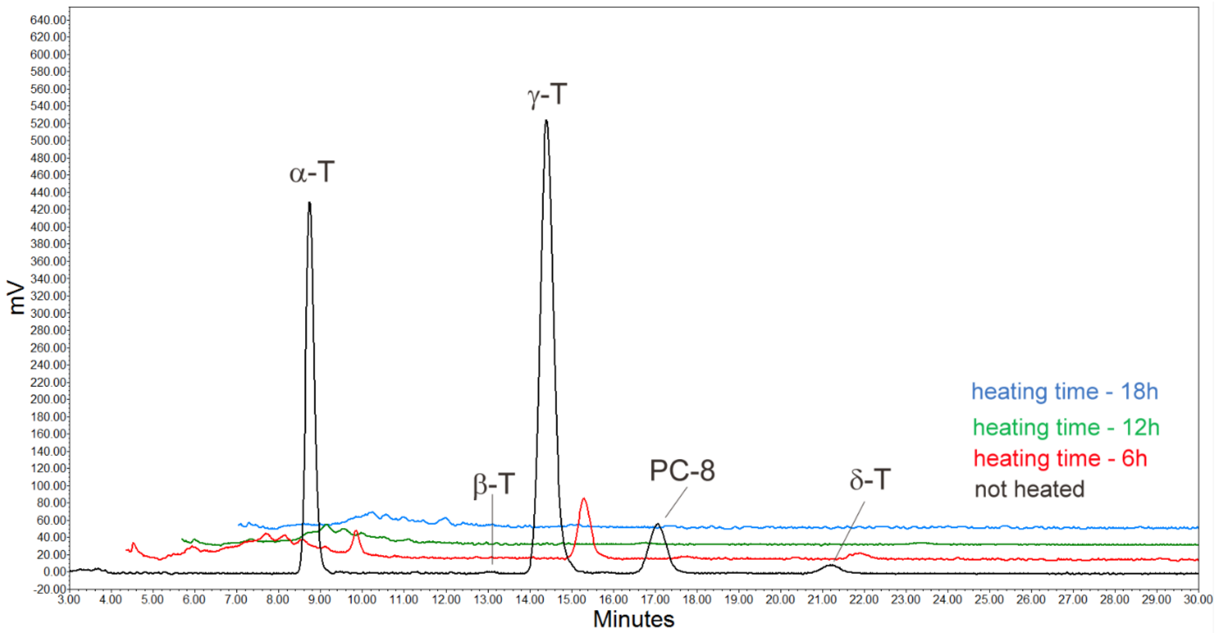

Figure S2. HPLC chromatogram of tocopherols composition changes in rapeseed oil heated at different times (6, 12, and 18 h).

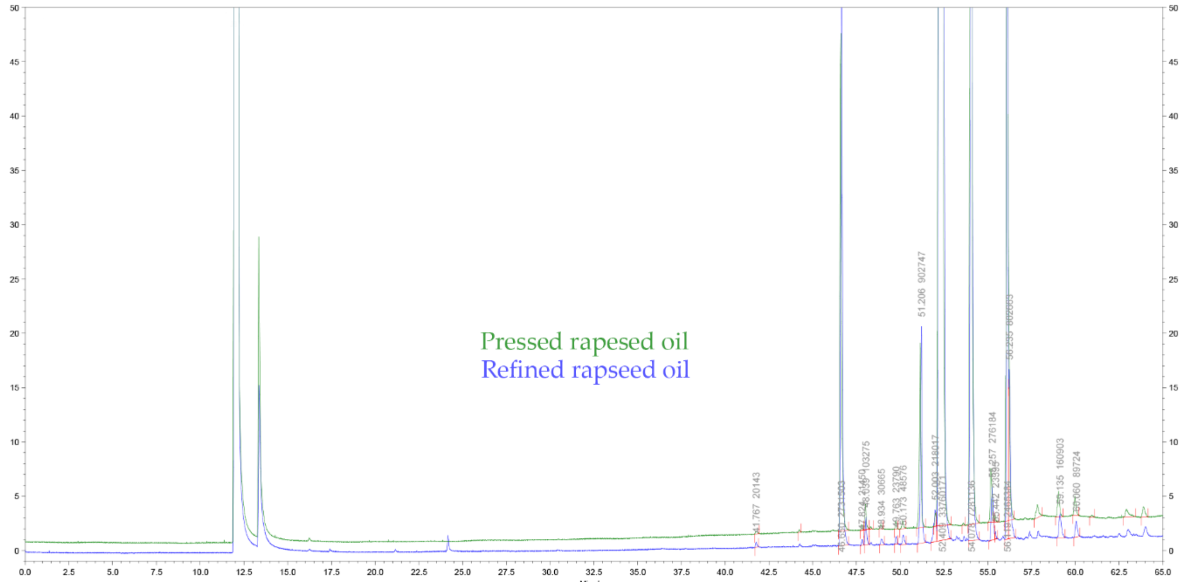

Figure S3. GC chromatogram of fresh pressed and refined rapeseed oil.
